# Evaluation of a Structured Educational Program for Community Pharmacists’ Knowledge, Attitudes, and Satisfaction Toward Diabetes Patient-Centered Care: A Non-Randomized Quasi-Experimental Study

**DOI:** 10.3390/healthcare14193260

**Published:** 2026-10-01

**Authors:** Heba Awadallah, Maher Khdour

**Affiliations:** 1 Faculty of Public Health, Al-Quds University, Jerusalem P.O. Box 20002, Palestine; heba.awadallah3@students.alquds.edu; 2 Faculty of Pharmacy, Al-Quds University, Jerusalem P.O. Box 20002, Palestine

**Keywords:** community pharmacists, educational intervention, knowledge, attitudes, satisfaction, patient-centered care

## Abstract

Background: Although diabetes is a major public health concern worldwide, evidence on formal diabetes education programs for community pharmacists in Palestine remains limited, despite their important role as accessible healthcare providers. Objectives: To evaluate immediate changes in community pharmacists’ diabetes-related knowledge and attitudes toward patient-centered care following participation in a structured diabetes education program and to assess their satisfaction with the educational experience. Methods: This non-randomized controlled pretest–posttest study enrolled 200 community pharmacists in the West Bank, Palestine. Knowledge and attitudes were assessed using modified versions of the Diabetes Knowledge Test and Diabetes Attitude Scale (DAS-3), respectively. Satisfaction with the educational experience was assessed after the educational period using an adapted questionnaire. General linear models provided adjusted post-intervention comparisons and between-group comparisons of pre–post changes. Results: At the pooled baseline knowledge mean, the adjusted post-intervention knowledge score was higher in the intervention group (adjusted mean difference = 6.73, 95% CI [6.13, 7.33], *p* < 0.001), and the adjusted change in knowledge from pre- to post-intervention was greater in the intervention group (adjusted mean difference in change = 7.04, 95% CI [6.37, 7.71], *p* < 0.001). At the pooled baseline attitude mean, the adjusted post-intervention attitude score was higher in the intervention group (adjusted mean difference = 0.579, 95% CI [0.393, 0.765], *p* < 0.001). The adjusted between-group difference in change in attitude was small and not statistically significant (adjusted mean difference in change = 0.091, 95% CI [−0.061, 0.242], *p* = 0.238). The adjusted satisfaction score was likewise higher in the intervention group (adjusted difference = 0.304, 95% CI [0.122, 0.487], *p* = 0.001). Conclusion: Participation in the structured educational program was associated with greater immediate knowledge gains and higher satisfaction than receipt of standard written materials. However, the adjusted between-group difference in attitude change was not statistically significant. The non-randomized design limits causal inference, and longer-term studies are needed to assess sustained educational benefits and changes in practice.

## 1. Introduction

In Palestine, diabetes mellitus is a serious and expanding public health issue that places a substantial burden on the Palestinian healthcare system [1,2]. However, there is still a lack of systematic integration of community pharmacist-led and CPD-supported models of treatment in the Palestinian setting, despite data from around the world suggesting that they can enhance diabetes outcomes [2,3,4]. The prevalence of diabetes among adult Palestinians increased from 10–12% in 2000 to 15% in 2010, and by 2020–2030, it is expected to reach 21–23% [5]. In the West Bank, 13.7% of primary-care patients already have prediabetes, which is closely associated with obesity, advanced age, male sex, inactivity, and poor diet [6]. Both microvascular and macrovascular problems are common, and only 16% of a large T2DM group had HbA1c < 7% [7]. Diabetes is a major cause of death and significantly reduces quality of life, but adherence to treatment and recommended clinical care remains suboptimal [8].

Patient-centered care, which emphasizes cooperative, comprehensive support that matches therapy to patients’ needs, preferences, and daily lives, has transformed contemporary health systems. As health centers become more accessible, community pharmacists are becoming increasingly important in providing this kind of care in collaboration with larger healthcare teams [9]. Using community pharmacists’ clinical knowledge in organized, team-based approaches offers a chance to improve outcomes and system effectiveness. The patient, not the product, is at the center of pharmaceutical care and person-centered pharmacy, which focuses on optimizing medication use, preventing drug-related issues, and improving quality of life [10,11]. Medication reviews, counseling, adherence support, screening, and health promotion are among the community pharmacist-led interventions that regularly improve clinical and behavioral outcomes as well as satisfaction and quality of life [12]. Despite evidence, role ambiguity, a lack of shared records, reimbursement obstacles, workload, and communication gaps continue to hinder collaboration [13]. Transitional care, collaborative diabetes treatment, deprescribing, and opioid-dependence care are examples of well-crafted pharmacist-led interventions that are possible, acceptable, and can improve safety, self-management, and quality of life, according to recent research [14].

To support patient-centered diabetes care in the community setting, structured educational interventions may help strengthen pharmacists’ knowledge and preparedness for expanded clinical roles. Therefore, this study aimed to evaluate a structured educational intervention designed to improve community pharmacists’ knowledge and attitudes toward patient-centered diabetes care and to assess their satisfaction with the program immediately after its delivery in the West Bank, Palestine.

## 2. Materials and Methods

### 2.1. Study Setting

We conducted the study among community pharmacists in the West Bank, Palestine, in April 2026. Community pharmacies are common in the West Bank and serve as accessible providers for patients with diabetes mellitus. The educational and all study assessments were delivered online so that pharmacists from different West Bank governorates could enroll.

### 2.2. Study Design

This was an open-label, non-randomized controlled quasi-experimental pretest-posttest study comparing a structured, live educational program with standard written educational materials. We enrolled 200 community pharmacists and allocated them through a non-random, availability-based procedure to the intervention group (*n* = 100) or control group (*n* = 100). We assessed knowledge and attitudes at baseline and immediately after the educational period, and satisfaction with the educational experience afterward. Participants and investigators were aware of group assignment. The study was reported in accordance with the Transparent Reporting of Evaluations with Nonrandomized Designs (TREND) statement.

### 2.3. Participants

Licensed pharmacists currently practicing in community pharmacies in the West Bank were eligible if they were employed in a community pharmacy, had internet access and an electronic device, and provided electronic informed consent. Availability to attend all five synchronous sessions determined allocation to the intervention group and was not an eligibility requirement for participation.

We recruited community pharmacists through professional pharmacist networks, direct contacts with community pharmacies, and electronic communication channels. According to the Palestinian Pharmacists Syndicate, 5841 pharmacists were registered in the West Bank in 2023 [15].

### 2.4. Intervention Group

Participants allocated to the intervention group attended a structured Diabetes Care Educational Program delivered through five live, synchronous Zoom sessions (Zoom Communications, Inc., San Jose, CA, USA) (Supplementary Files Tables S1–S3). A pharmacist with professional and academic experience in pharmacotherapy and diabetes care delivered the program. It consisted of five structured modules covering 22 subtopics: diabetes classification, pathophysiology, risk factors, diagnosis, laboratory findings, and therapeutic principles; glycemic targets, nutrition, physical activity, weight management, and lifestyle modification; selection, mechanisms of action, adverse effects, contraindications, and monitoring of oral glucose-lowering medications; insulin types, dosing regimens, administration, storage, monitoring, hypoglycemia, and diabetic ketoacidosis; and diabetes management in pregnancy and older adults, together with microvascular and macrovascular complications, including retinopathy, nephropathy, neuropathy, cardiovascular disease, and diabetic foot care.

The sessions included PowerPoint presentations, case-based discussions, clinical scenarios, interactive questions, and question-and-answer activities. These methods supported applying diabetes knowledge to community-pharmacy practice.

Attendance was monitored throughout the program. To reduce contamination between the study groups, intervention participants were asked not to share educational materials with control participants until post-intervention data collection was complete.

### 2.5. Control Group

Participants allocated to the control group received standard written educational materials on diabetes care, such as publicly available guidelines, brochures, or informational documents. The materials covered general diabetes information but did not include the structured curriculum, live teaching, clinical cases, interactive discussions, or question-and-answer activities provided to the intervention group. Both groups completed the same knowledge and attitude assessments at baseline and immediately after the study period.

### 2.6. Sample Size

The sample size was estimated based on the expected between-group difference in the primary outcome of diabetes knowledge. Assuming a medium effect size (Cohen’s d = 0.50), a two-sided significance level of 5%, and 80% power to detect a meaningful difference, 64 participants were required in each group. To account for an anticipated non-response or loss-to-follow-up rate of approximately 36%, the target sample size was increased to 100 pharmacists per group, resulting in a total sample of 200 participants.

### 2.7. Group Allocation

Participants were allocated using a non-random, availability-based procedure. Pharmacists who confirmed their availability and willingness to attend all five synchronous Zoom sessions were assigned to the intervention group, whereas eligible pharmacists who could not attend the scheduled sessions were assigned to the control group. Therefore, this procedure represents convenience/self-selection allocation and did not provide each participant with an equal probability of assignment to either group.

### 2.8. Outcomes

The primary outcomes were changes in pharmacists’ diabetes-related knowledge and attitudes from baseline to immediately after the intervention. The secondary outcome was participants’ satisfaction with the educational experience.

### 2.9. Knowledge Test

A modified version of the University of Michigan Diabetes Research and Training Center (MDRTC) Diabetes Knowledge Test (DKT) was used to assess community pharmacists’ understanding of diabetes management [16]. The modified DKT comprised 17 multiple-choice items across four subdomains. Each correct response was scored 1, and each incorrect response was scored 0, yielding a total knowledge score ranging from 0 to 17. A research participant receives a score of “1” for each correct response and a score of “0” for each incorrect response.

### 2.10. Attitude Test

A modified version of the Diabetes Attitude Scale (DAS-3) was used [17,18] to assess community pharmacists’ attitudes toward managing and monitoring diabetes and its consequences. At both time points, participants scored statements on the DAS-3 questionnaire on a 5-point Likert scale. Questions 1–4, 8, and 10–12 of the DAS-3 scale’s 12 attitude questions use values ranging from “5” for strong agreement to “1” for extreme disagreement, whereas Items 5–7 and 9 were reverse-scored so that higher scores consistently indicated more favorable attitudes toward diabetes care. Higher attitude scores indicate a more favorable attitude toward diabetes care among community pharmacists. Although the DKT and DAS-3 scales had previously been validated and pre-tested, an expert panel comprising a diabetologist, a clinical pharmacist, and an academic and researcher with expertise in survey-based questionnaire development retested the modified local version for suitability and reliability. To assess the questionnaire’s usability, flow, layout, and clarity, ten community pharmacists conducted pilot testing.

### 2.11. Satisfaction Test

The tool was adapted from validated frameworks used in continuing professional education research and modified from validated methods in the literature on pharmacist education to ensure content validity [19]. The instrument covers two primary domains: overall satisfaction with the session’s organization and execution, and perceived influence on professional practice, including confidence and drive for further learning, with responses rated on a 5-point Likert scale.

### 2.12. Instrument Validation and Reliability

The knowledge and attitude instruments had been validated in previous studies. Nevertheless, Cronbach’s alpha was calculated to assess their internal consistency in the Palestinian community-pharmacy setting. Reliability coefficients indicated acceptable to excellent internal consistency in Table 1. Reliability coefficients were 0.841 and 0.838 for baseline and post-intervention knowledge, respectively; 0.773 and 0.732 for baseline and post-intervention attitude, respectively; and 0.927 for satisfaction, indicating acceptable to excellent reliability.

### 2.13. Data Collection Procedure

Data were collected via Google Forms at two time points (Google LLC, Mountain View, CA, USA), before group exposure and directly after the intervention program ended. Prior to enrolment, participants were informed of study purpose, procedures, possible benefits, voluntary participation and confidentiality. Pharmacists who met eligibility criteria electronically consented prior to accessing the baseline questionnaire. The sociodemographic and occupational variables collected by the baseline questionnaire were age, gender, educational qualification, years of professional experience, years of practice in a community pharmacy, and the knowledge and attitude instruments. At the end of the education period, both groups completed knowledge and attitude questionnaires.

### 2.14. Data Analysis

Data were analyzed using IBM SPSS Statistics version 27 (IBM Corp., Armonk, NY, USA). Frequencies and percentages summarized categorical variables, while means and standard deviations summarized continuous outcomes. We compared baseline sociodemographic and professional characteristics between groups using Pearson’s chi-square tests. We compared unadjusted post-intervention knowledge, attitude, and satisfaction scores using independent-samples t tests. We examined within-group pre-post changes in knowledge and attitude using paired-samples t tests. We explored differences in post-intervention outcomes across sociodemographic and professional categories using independent-samples t tests or one-way analysis of variance, as appropriate. We reported mean differences, 95% confidence intervals, and Cohen’s d effect sizes for unadjusted comparisons.

Because allocation was non-random and the groups differed significantly in age and gender, we performed adjusted between-group analyses using general linear models with HC3 heteroskedasticity-consistent standard errors. We adjusted post-intervention total knowledge and attitude scores for their corresponding baseline scores, gender, and age group. We evaluated the homogeneity-of-regression-slopes assumption using group-by-baseline interaction terms. We identified significant interactions for knowledge and attitude; therefore, we retained these terms and estimated conditional group comparisons at the respective pooled baseline means. Satisfaction, which was measured only after the educational period, was adjusted for gender and age group without a baseline satisfaction covariate. As a complementary analysis directly addressing differential change, we compared change scores (post-test minus pre-test) between groups after adjustment for gender and age group. Adjusted differences, HC3 robust standard errors, 95% confidence intervals, *p*-values, and partial eta-squared were reported. We set statistical significance at *p* < 0.05.

## 3. Results

**Figure 1:**
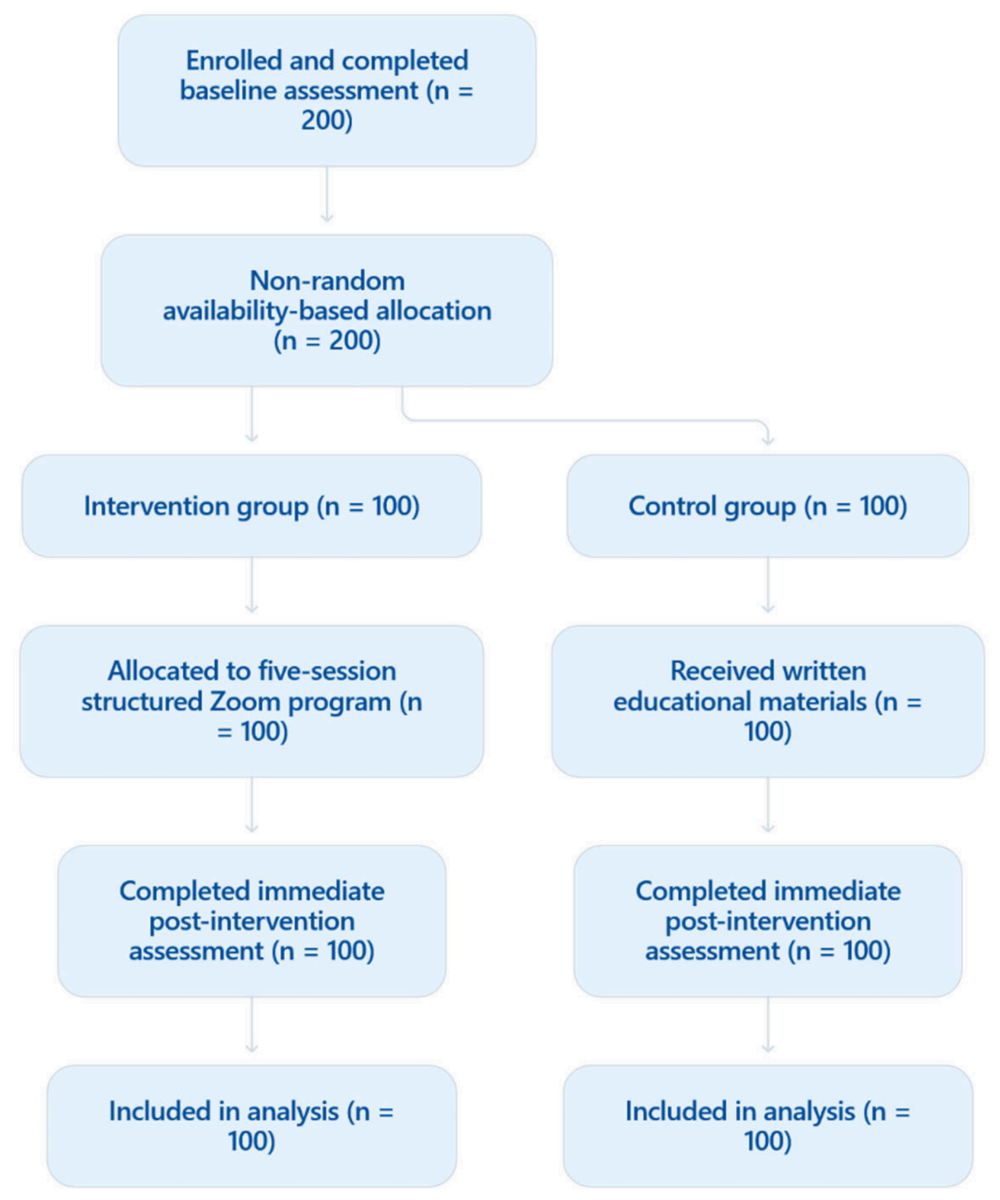
Participant flow through enrolment, non-random availability-based allocation, intervention delivery, immediate post-intervention assessment, and analysis.

We enrolled and allocated 200 eligible community pharmacists using a non-random, availability-based procedure to the intervention group (*n* = 100) or control group (*n* = 100). All participants completed the baseline and immediate post-intervention assessments and were included in the final analysis (*n* = 100 per group), as shown in Figure 1.

### 3.1. Comparison of Baseline Sociodemographic and Professional Characteristics Between the Intervention and Control Groups

Table 2 presents the baseline sociodemographic and professional characteristics; the intervention and control groups were compared to assess group equivalence before the educational intervention (Table 2). Significant differences were observed between the two groups in terms of gender, χ^2^(1, *n* = 200) = 6.67, *p* = 0.010, and age group, χ^2^(3, *n* = 200) = 10.94, *p* = 0.012. The intervention group included a higher proportion of male pharmacists (29.0%) than the control group (14.0%), while females made up the majority in both groups (71.0% vs. 86.0%, respectively). In addition, participants in the intervention group were generally older, with a greater proportion aged 31–40 years (38.0% vs. 20.0%) compared with the control group. No statistically significant differences were found between the intervention and control groups regarding years of professional experience, χ^2^(3, *n*= 200) = 6.82, *p* = 0.078, educational qualification, χ^2^(3, N = 200) = 6.39, *p* = 0.094, or years of practice in community pharmacy in Palestine, χ^2^(3, N = 200) = 1.46, *p* = 0.692.

### 3.2. Unadjusted Post-Intervention Comparison of Knowledge, Attitude, and Satisfaction Scores Between the Intervention and Control Groups

Following the intervention, independent-samples t tests compared post-intervention knowledge, attitude, and satisfaction scores between the intervention and control groups (Table 3). The intervention group demonstrated significantly higher overall knowledge sum scores (M = 14.56, SD = 2.39) than the control group (M = 8.05, SD = 2.53), t(198) = 18.70, *p* < 0.001, d = 2.65, representing a large standardized mean difference.

Analysis of the knowledge subdomains showed that the intervention group significantly outperformed the control group across all domains. Specifically, participants in the intervention group scored higher on nutrition knowledge (M = 5.95, SD = 1.28) than the control group (M = 4.34, SD = 1.65), t(198) = 7.72, *p* < 0.001, d = 1.09. Similarly, oral medication knowledge was substantially higher in the intervention group (M = 2.82, SD = 0.39) compared with the control group (M = 1.26, SD = 0.63), t(198) = 21.12, *p* < 0.001, d = 2.99. Participants receiving the intervention also scored significantly higher on insulin knowledge (M = 3.26, SD = 0.86 vs. M = 1.32, SD = 0.93), t(198) = 15.31, *p* < 0.001, d = 2.17, and complication knowledge (M = 2.53, SD = 0.67 vs. M = 1.13, SD = 0.53), t(198) = 16.39, *p* < 0.001, d = 2.32. The effect sizes for these knowledge domains were all large, indicating substantial improvements associated with the intervention.

The intervention group also reported significantly more positive attitudes toward diabetes management (M = 4.11, SD = 0.50) than the control group (M = 3.28, SD = 0.56), t(198) = 11.08, *p* < 0.001, d = 1.57, reflecting a large effect. Likewise, participant satisfaction was significantly higher in the intervention group (M = 4.35, SD = 0.66) compared with the control group (M = 3.99, SD = 0.53), t(198) = 4.27, *p* < 0.001, d = 0.60, corresponding to a medium effect size.

### 3.3. Pre- and Post-Intervention Comparison of Knowledge and Attitude Scores in the Intervention Group

Paired-samples *t* tests were conducted to evaluate changes in knowledge and attitude scores from pre-test to post-test within the intervention group (Table 4). The results demonstrated significant improvements across all measured outcomes following the educational intervention. Overall knowledge scores increased significantly from pre-test (M = 7.12, SD = 1.22) to post-test (M = 14.56, SD = 2.39), t(99) = 27.09, *p* < 0.001, d = 2.71, indicating a large standardized mean difference.

The intervention group had significantly higher unadjusted post-intervention scores across all knowledge subdomains. Nutrition knowledge increased from M = 3.43 (SD = 0.86) at baseline to M = 5.95 (SD = 1.28) following the intervention, t(99) = 16.50, *p* < 0.001, d = 1.65. Oral medication knowledge improved from M = 1.19 (SD = 0.44) to M = 2.82 (SD = 0.39), t(99) = 26.55, *p* < 0.001, d = 2.66. Similarly, insulin knowledge increased from M = 1.17 (SD = 0.51) to M = 3.26 (SD = 0.86), t(99) = 21.43, *p* < 0.001, d = 2.14, whereas complication knowledge improved from M = 1.33 (SD = 0.47) to M = 2.53 (SD = 0.67), t(99) = 13.52, *p* < 0.001, d = 1.35. All knowledge domains showed large effect sizes, with the greatest improvement in oral medication knowledge.

Participants also exhibited a modest but statistically significant improvement in attitude scores, increasing from M = 3.96 (SD = 0.28) at pre-test to M = 4.11 (SD = 0.50) at post-test, t(99) = 2.76, *p* = 0.007, d = 0.28, representing a small effect size.

### 3.4. Pre- and Post-Intervention Comparison of Knowledge and Attitude Scores in the Control Group

Paired-samples t tests were conducted to examine changes in knowledge and attitude scores from pre-test to post-test within the control group (Table 5). Overall, total knowledge and most knowledge subdomains did not improve significantly over the study period. Oral medication knowledge improved significantly but modestly, increasing from M = 1.11 (SD = 0.42) at baseline to M = 1.26 (SD = 0.63) at post-test, t(99) = 2.61, *p* = 0.011, d = 0.26. Attitude scores also improved modestly from M = 3.16 (SD = 0.43) to M = 3.28 (SD = 0.56), t(99) = 2.37, *p* = 0.020, d = 0.24, representing a small effect size.

### 3.5. Differences Between Participant Characteristics and Post-Test Knowledge Scores

Independent-samples t tests and one-way analyses of variance (ANOVAs) examined differences in post-test knowledge scores by participants’ sociodemographic and professional characteristics (Table 6). No statistically significant differences were observed across any of the examined characteristics.

Post-test knowledge scores did not differ significantly according to gender, t(59.32) = 1.81, *p* = 0.076; age group, F(3, 196) = 1.00, *p* = 0.392; years of professional experience, F(3, 196) = 1.20, *p* = 0.313; educational qualification, F(3, 196) = 0.45, *p* = 0.718; or years of practice in community pharmacy, F(3, 196) = 0.48, *p* = 0.699.

Overall, these findings indicate that post-test diabetes knowledge was comparable across participants regardless of gender, age, professional experience, educational qualification, or years of practice in community pharmacy, suggesting that these demographic and professional characteristics were not significant determinants of knowledge outcomes in the study population.

### 3.6. Differences Between Participant Characteristics and Post-Test Attitude Scores

Post-test attitude scores did not differ significantly according to gender, *t*(198) = 1.65, *p* = 0.100, age group, *F*(3, 196) = 0.65, *p* = 0.584, years of professional experience, *F*(3, 196) = 0.65, *p* = 0.582, educational qualification, *F*(3, 196) = 1.09, *p* = 0.355, or years of practice in community pharmacy, *F*(3, 196) = 2.56, *p* = 0.056. These findings indicate that post-test attitude scores were comparable across all participant characteristics in Table 7.

### 3.7. Association Between Participant Characteristics and Post-Test Satisfaction Scores

Independent-samples t tests and one-way analyses of variance (ANOVAs) were conducted to examine differences in post-test satisfaction scores according to participants’ sociodemographic and professional characteristics (Table 8). Post-test satisfaction scores differed significantly by age group, F(3, 196) = 2.96, *p* = 0.034, with participants aged 31–40 years reporting the highest satisfaction (M = 4.36, SD = 0.48) according to the Tukey post hoc test. However, satisfaction scores did not differ significantly according to gender, t(198) = 1.49, *p* = 0.139, years of professional experience, F(3, 196) = 1.63, *p* = 0.183, educational qualification, F(3, 196) = 1.24, *p* = 0.295, or years of practice in community pharmacy, F(3, 196) = 2.30, *p* = 0.078.

### 3.8. Floor and Ceiling Effects

Post-intervention score distributions showed no substantial floor effects, with percentages ranging from 0% to 8%. In contrast, the intervention group showed ceiling effects for all knowledge outcomes: total knowledge (25%), nutrition knowledge (46%), oral-medication knowledge (82%), insulin knowledge (48%), and complication knowledge (63%). The most pronounced ceiling effect occurred for oral-medication knowledge, with all intervention-group scores ranging from 2 to 3 and 82% of participants achieving the maximum score. In the control group, a ceiling effect was observed only for nutrition knowledge (19%). These restricted score ranges and reduced post-intervention variability may have contributed to the magnitude of the standardized differences.

### 3.9. Adjusted Between-Group Comparisons of Post-Intervention Outcomes and Pre–Post Changes

Adjusted between-group analyses are presented in Table 9. Significant group-by-baseline interactions were identified for total knowledge and attitude; therefore, these interactions were retained, and conditional comparisons were estimated at the respective pooled baseline means. At the mean baseline knowledge score of 7.405, the intervention group had a significantly higher adjusted post-intervention knowledge score than the control group, adjusted difference = 6.73, robust *SE* = 0.30, 95% CI [6.13, 7.33], *t*(192) = 22.22, *p* < 0.001, partial η^2^ = 0.720. At the mean baseline attitude score of 3.5598, the adjusted post-intervention difference was also significant, adjusted difference = 0.579, robust *SE* = 0.094, 95% CI [0.393, 0.765], *t*(192) = 6.14, *p* < 0.001, partial η^2^ = 0.164.

Satisfaction, which was measured only after the intervention, remained significantly higher in the intervention group after adjustment for gender and age group, adjusted difference = 0.304, robust *SE* = 0.093, 95% CI [0.122, 0.487], *t*(194) = 3.29, *p* = 0.001, partial η^2^ = 0.053.

Direct comparisons of change showed that knowledge increased significantly more in the intervention group, adjusted difference = 7.04, robust *SE* = 0.34, 95% CI [6.37, 7.71], *t*(194) = 20.77, *p* < 0.001, partial η^2^ = 0.690. In contrast, the adjusted difference in attitude change was small and nonsignificant, adjusted difference = 0.091, robust *SE* = 0.077, 95% CI [−0.061, 0.242], *t*(194) = 1.18, *p* = 0.238, partial η^2^ = 0.007. Thus, the large conditional post-test attitude difference did not correspond to a significantly greater improvement in attitude over time (Table 9).

## 4. Discussion

The results of this non-randomized quasi-experimental study indicate that the structured education program was associated with a greater immediate increase in diabetes patient-centered care knowledge among community pharmacists in Palestine. However, interpretation of comparisons between the intervention and control group should be made with appropriate caution in light of allocation by availability, imbalances in baseline characteristics, and selection bias and residual confounding. Our findings suggest an association between participation in structured training and greater immediate diabetes knowledge; changes in pharmacists’ clinical roles or practice were not assessed [20].

However, the higher post-intervention attitude score should be interpreted in the context of the substantial baseline difference in attitude, as the adjusted between-group difference in attitude change was not statistically significant(*p* < 0.001, d = 2.65) and attitude scores (*p* < 0.001, d = 1.57) compared with the control group. This finding is consistent with other studies conducted in the region. For instance, a study in Saudi Arabia found that community pharmacists who received a structured educational session on diabetes experienced a notable increase in their understanding of oral hypoglycemic medications, disease monitoring, and insulin dosing (*p* = 0.01). Their attitude scores also improved, rising from 49.74 to 52.74. In the present study, large unadjusted standardized differences were observed across knowledge domains, alongside a significant adjusted between-group difference in knowledge change [21]. Similarly, research from Jordan found that although most community pharmacists had an intermediate level of knowledge (72.3%) and high practice levels (58.1%) regarding diabetes management during Ramadan, notable deficiencies remained in areas such as medication adjustments and patient risk stratification [8].

The significant improvements in knowledge of insulin (d = 2.17) and oral medications (d = 2.99) are particularly noteworthy, as these are areas where pharmacists can most directly influence patient outcomes through effective counseling and medication management. A study conducted in the UAE found that approximately 75% of community pharmacists offer guidance on insulin administration and the timing of oral antidiabetic drugs, yet knowledge deficiencies remain prevalent. The higher knowledge scores observed among intervention participants suggest potential educational value, although changes in counseling practices and patient outcomes were not assessed [20].

The baseline age and gender differences indicate the need for adjusted analyzes, as there were more male pharmacists and more 31–40 year old participants in the intervention group. Demographic adjustment does not account for differences in motivation, interest in diabetes care prior to the program, willingness to participate in a continuing medical education intervention, or unmeasured confounding by self-selection. Similarly, nonsignificant exploratory associations between demographic characteristics at baseline. The exploratory demographic comparisons do not establish that the association between program participation and outcomes was consistent across participant subgroups. Interactions between group allocation and demographic characteristics were not evaluated; therefore, similar benefits across demographic groups cannot be assumed. Interestingly, a Saudi Arabian study found that pharmacists with bachelor’s degrees and those between the ages of 30 and 39 tended to have higher average scores on diabetes knowledge [22]. Our post-hoc study showed that demographic characteristics had no discernible effect on the post-test findings, despite these initial demographic differences. This suggests that pharmacists from different backgrounds found the intervention successful. Practically, this suggests the intervention is broadly applicable, regardless of the participating pharmacists’ demographics.

There was a large and statistically significant adjusted difference in change in knowledge. The adjusted difference in change in attitude was small and not statistically Significant. While the intervention group came out considerably higher than the control group on an adjusted post-intervention attitude score when that score was conditioned on the pooled baseline mean, this difference did not translate to more improvement over time. The attitude differences between groups at baseline likely contributed to the differences between groups after intervention, and should be taken into account when interpreting the current results in the context of studies in the region that showed attitude improvements with pharmacist education.

The large unadjusted standardized differences in insulin and oral-medication knowledge require cautious interpretation. These subdomains contained few dichotomously scored items and showed upper-end clustering, with 82% of intervention participants achieving the maximum oral-medication score. Restricted score ranges and reduced within-group variability may have contributed to the magnitude of the standardized differences. These findings indicate differences in test performance but do not establish corresponding improvements in clinical practice or patient outcomes. These subdomains had very few dichotomously scored items subject to ceiling effects, including oral-medication knowledge, where 82% of intervention participants achieved the maximum score.

In comparison, the intervention group had a more favorable attitude toward diabetes self-management than the control group (M = 4.11, SD = 0.50 vs. M = 3.28, SD = 0.56; *p* < 0.001, d = 1.57). In the Sudan study, the improvement in mean attitude from baseline was equal to 1.7 (±0.2, *p* < 0.001) [23]. Although attitude scores improved modestly within the intervention group (pre–post d = 0.28, *p* = 0.007), this within-group change should not be interpreted as evidence of an intervention-specific effect because the adjusted between-group difference in attitude change was not statistically significant. However, literature has shown that “high knowledge scores about diabetes will not improve attitudes and practices related to diabetes” when there have been no specific interventions in those areas [23]. Although the intervention group showed a small within-group increase and had a higher adjusted post-intervention score at the pooled baseline mean, the adjusted between-group difference in attitude change was not statistically significant. Consequently, the study does not provide evidence that participation in the program produced a greater improvement in attitude than the comparison condition.

Interestingly, In exploratory analyses, satisfaction scores differed across age groups (*p* = 0.034), with participants aged 31–40 years having the highest observed mean. This finding should be interpreted cautiously because these comparisons did not adjust for study group or other characteristics. Overall, the intervention group reported higher satisfaction after adjustment for gender and age group. These findings suggest that focused educational initiatives may help address perceived knowledge gaps while providing a positively evaluated continuing professional development experience. Earlier studies have identified a lack of time (68.7%), insufficient knowledge or clinical skills (68.7%), and poor cooperation with other healthcare professionals (77%) as significant barriers to pharmacists delivering diabetes care [20]. Our findings suggest that focused educational initiatives to close these knowledge gaps may increase pharmacists’ self-assurance and overall satisfaction with their clinical duties.

However, the study has its strengths, notably the pre-test-posttest Non-randomized controlled design, adequate sample size, ability to recruit pharmacists from different regions of the West Bank, and acceptable local reliability of the instruments used. The study also exhibits some limitations. Allocation to the groups was not randomized; participants interested in the intervention were required to be able and willing to attend the five synchronous sessions. Therefore, intervention participants may have been systematically different from control participants on motivation, diabetes care interest, willingness to engage in continuing education, or other factors. Self-selection bias may limit this study’s ability to show causality. While analyzes were adjusted for baseline values, age, and gender, residual confounding cannot be completely excluded. Outcomes were measured at the end of the education intervention. It is unknown if this was sustained knowledge retention, long-term change in attitudes, or change in practice. Because of the open-label design, there may have also been response bias and social-desirability bias in attitudes and satisfaction measures. Blinding was not possible as participants knew whether they had attended the live sessions or not, and no social-desirability measures were employed. Electronic data collection with no face to face contact with the researcher and confidentiality guarantees may have reduced this risk, but it could not be eliminated. No assessment was made of whether the changes in education activities were accompanied by a change in counseling and diabetes-related behavior, glycemic control, medication adherence, or other patient-related outcome. Future randomized trials should be conducted with longer follow-up periods, and include objective measures of professional practice and patient outcomes. The findings represent immediate educational changes and do not establish long-term knowledge retention, sustained changes in attitudes, or changes in pharmacists’ professional behavior. Furthermore, the study did not determine whether the observed educational changes translated into improvements in medication counseling, diabetes-care practices, glycemic control, medication adherence, or other patient-level outcomes. Future studies should include multiple follow-up assessments and objective measures of professional practice and patient outcomes.

## 5. Conclusions

Participation in the structured diabetes education program was associated with greater immediate improvements in community pharmacists’ knowledge of patient-centered diabetes care and higher satisfaction compared with standard written educational materials. Although the intervention group had a higher adjusted post-intervention attitude score, the adjusted between-group difference in attitude change was not statistically significant; therefore, the findings do not support a greater improvement in attitudes attributable to the program. Given the non-randomized allocation, baseline group differences, and immediate post-intervention assessment, the findings should be interpreted cautiously. Further randomized studies with longer follow-up and objective measures of professional practice and patient outcomes are needed to determine whether these immediate educational gains are sustained and translate into improved diabetes care.

## Figures and Tables

**Table 1:** Internal Consistency Reliability of the Study Instruments.

Instrument	No. of Items	Cronbach’s α
Pre-test knowledge sum score	17	0.841
Post-test knowledge sum score	17	0.838
Pre-test attitude mean score	12	0.773
Post-test attitude mean score	12	0.732
Post-test satisfaction mean score	8	0.927

**Table 2:** Comparison of Baseline Sociodemographic and Professional Characteristics Between the Intervention and Control Groups (*n* = 200).

Variable	Category	Intervention (*n* = 100) *n* (%)	Control (*n* = 100) *n* (%)	χ^2^ (df)	*p*
Gender	Male	29 (29.0)	14 (14.0)	6.67 (1)	0.010 *
	Female	71 (71.0)	86 (86.0)		
Age Group	21–25 years	18 (18.0)	33 (33.0)	10.94 (3)	0.012 *
	26–30 years	22 (22.0)	28 (28.0)		
	31–40 years	38 (38.0)	20 (20.0)		
	>40 years	22 (22.0)	19 (19.0)		
Years of Experience	1–3 years	27 (27.0)	42 (42.0)	6.82 (3)	0.078
	3–6 years	22 (22.0)	24 (24.0)		
	6–10 years	21 (21.0)	15 (15.0)		
	>10 years	30 (30.0)	19 (19.0)		
Educational Qualification	Bachelor of Pharmacy (B. Pharm)	62 (62.0)	55 (55.0)	6.39 (3)	0.094
	Doctor of Pharmacy (Pharm. D.)	12 (12.0)	8 (8.0)		
	Master’s Degree in Pharmacy	18 (18.0)	17 (17.0)		
	PhD	8 (8.0)	20 (20.0)		
Years of Practice in Community Pharmacy in Palestine	1–3 years	34 (34.0)	39 (39.0)	1.46 (3)	0.692
	3–6 years	29 (29.0)	22 (22.0)		
	6–10 years	12 (12.0)	14 (14.0)		
	>10 years	25 (25.0)	25 (25.0)		

Note. χ^2^ = Pearson’s chi-square statistic; df = degrees of freedom. * p ≤ 0.05.

**Table 3:** Unadjusted Post-Intervention Comparison of Knowledge, Attitude, and Satisfaction Scores Between the Intervention and Control Groups.

Outcome	Intervention M ± SD	Control M ± SD	Mean Difference (95% CI)	*t*(df)	*p*	Cohen’s *d*
Total knowledge score	14.56 ± 2.39	8.05 ± 2.53	6.51 (5.82, 7.20)	18.70 (198)	<0.001 *	2.65
Nutrition knowledge	5.95 ± 1.28	4.34 ± 1.65	1.61 (1.20, 2.02)	7.72 (198)	<0.001 *	1.09
Oral medication knowledge	2.82 ± 0.39	1.26 ± 0.63	1.56 (1.41, 1.71)	21.12 (198)	<0.001 *	2.99
Insulin knowledge	3.26 ± 0.86	1.32 ± 0.93	1.94 (1.69, 2.19)	15.31 (198)	<0.001 *	2.17
Complication knowledge	2.53 ± 0.67	1.13 ± 0.53	1.40 (1.23, 1.57)	16.39 (198)	<0.001 *	2.32
Attitude score	4.11 ± 0.50	3.28 ± 0.56	0.83 (0.68, 0.98)	11.08 (198)	<0.001 *	1.57
Satisfaction score	4.35 ± 0.66	3.99 ± 0.53	0.36 (0.19, 0.53)	4.27 (198)	<0.001 *	0.60

*Note*. Values are presented as M ± SD. Independent-samples *t* tests compared post-intervention outcomes between the intervention and control groups. Cohen’s d was calculated using the pooled standard deviation and interpreted as 0.20 = small, 0.50 = medium, and 0.80 = large standardized mean difference. * p = < *0.05*.

**Table 4:** Pre- and Post-Intervention Comparison of Knowledge and Attitude Scores in the Intervention Group (*n* = 100).

Outcome	Pre-Test, M ± SD	Post-Test, M ± SD	Mean Difference (95% CI)	*t*(99)	*p*	Cohen’s *d*
Total knowledge score	7.12 ± 1.22	14.56 ± 2.39	7.44 (6.90, 7.98)	27.09	<0.001 *	2.71
Nutrition knowledge	3.43 ± 0.86	5.95 ± 1.28	2.52 (2.22, 2.82)	16.50	<0.001 *	1.65
Oral medication knowledge	1.19 ± 0.44	2.82 ± 0.39	1.63 (1.51, 1.75)	26.55	<0.001 *	2.66
Insulin knowledge	1.17 ± 0.51	3.26 ± 0.86	2.09 (1.90, 2.28)	21.43	<0.001 *	2.14
Complication knowledge	1.33 ± 0.47	2.53 ± 0.67	1.20 (1.02, 1.38)	13.52	<0.001 *	1.35
Attitude score	3.96 ± 0.28	4.11 ± 0.50	0.16 (0.04, 0.27)	2.76	0.007 *	0.28

***Note.*** Mean difference is reported as Post − Pre (absolute values shown for ease of interpretation). Cohen’s *d* represents the standardized mean difference for paired samples. * p = < *0.05*.

**Table 5:** Pre- and Post-Intervention Comparison of Knowledge and Attitude Scores in the Control Group (*n* = 100).

Outcome	Pre-Test, M ± SD	Post-test, M ± SD	Mean Difference (95% CI)	*t*(99)	*p*	Cohen’s *d*
Total knowledge score	7.69 ± 1.92	8.05 ± 2.53	0.36 (−0.02, 0.74)	1.90	0.060	0.19
Nutrition knowledge	4.28 ± 1.56	4.34 ± 1.65	0.06 (−0.07, 0.19)	0.95	0.345	0.10
Oral medication knowledge	1.11 ± 0.42	1.26 ± 0.63	0.15 (0.04, 0.26)	2.61	0.011 *	0.26
Insulin knowledge	1.16 ± 0.53	1.32 ± 0.93	0.16 (−0.03, 0.35)	1.67	0.099	0.17
Complication knowledge	1.14 ± 0.43	1.13 ± 0.53	−0.01 (−0.11, 0.09)	0.19	0.849	0.02
Attitude score	3.16 ± 0.43	3.28 ± 0.56	0.12 (0.02, 0.22)	2.37	0.020 *	0.24

***Note.*** Mean difference is reported as Post − Pre (absolute values shown for ease of interpretation). Cohen’s *d* represents the standardized mean difference for paired samples. * p = < *0.05*.

**Table 6:** Differences Between Participant Characteristics and Post-Test Knowledge Scores (*n* = 200).

Characteristic	Category	*n*	Mean ± SD	Test Statistic	*p*
Gender	Male	43	12.40 ± 4.61	*t*(59.32) = 1.81	0.076
	Female	157	11.01 ± 3.89		
Age Group	21–25 years	51	10.75 ± 4.00	*F*(3, 196) = 1.00	0.392
	26–30 years	50	11.00 ± 3.91		
	31–40 years	58	12.02 ± 4.38		
	>40 years	41	11.37 ± 3.95		
Years of Experience	1–3 years	69	10.99 ± 3.72	*F*(3, 196) = 1.20	0.313
	3–6 years	46	10.67 ± 3.96		
	6–10 years	36	11.67 ± 4.42		
	>10 years	49	12.08 ± 4.41		
Educational Qualification	B. Pharm	117	11.37 ± 3.80	*F*(3, 196) = 0.45	0.718
	Pharm. D.	20	11.90 ± 4.53		
	Master’s	35	11.34 ± 4.91		
	PhD	28	10.57 ± 3.91		
Years of Practice in Community Pharmacy	1–3 years	73	11.05 ± 3.97	*F*(3, 196) = 0.48	0.699
	3–6 years	51	11.82 ± 3.72		
	6–10 years	26	10.85 ± 4.62		
	>10 years	50	11.38 ± 4.37		

*Note.* Independent-samples t tests and one-way tests.

**Table 7:** Differences Between Participant Characteristics and Post-Test Attitude Scores (*n* = 200).

Characteristic	Category	*n*	Mean ± SD	Test Statistic	*p*
Gender	Male	43	3.85 ± 0.60	*t*(198) = 1.65	0.100
	Female	157	3.66 ± 0.69		
Age Group	21–25 years	51	3.72 ± 0.71	*F*(3, 196) = 0.65	0.584
	26–30 years	50	3.61 ± 0.69		
	31–40 years	58	3.78 ± 0.66		
	>40 years	41	3.66 ± 0.62		
Years of Experience	1–3 years	69	3.75 ± 0.72	*F*(3, 196) = 0.65	0.582
	3–6 years	46	3.58 ± 0.64		
	6–10 years	36	3.74 ± 0.69		
	>10 years	49	3.71 ± 0.62		
Educational Qualification	B. Pharm	117	3.74 ± 0.64	*F*(3, 196) = 1.09	0.355
	Pharm. D.	20	3.78 ± 0.72		
	Master’s	35	3.69 ± 0.75		
	PhD	28	3.49 ± 0.68		
Years of Practice in Community Pharmacy	1–3 years	73	3.70 ± 0.69	*F*(3, 196) = 2.56	0.056
	3–6 years	51	3.87 ± 0.60		
	6–10 years	26	3.44 ± 0.82		
	>10 years	50	3.65 ± 0.60		

*Note.* Independent-samples *t* tests and one-way tests.

**Table 8:** Association Between Participant Characteristics and Post-Test Satisfaction Scores (*n* = 200).

Characteristic	Category	*n*	Mean ± SD	Test Statistic	*p*
Gender	Male	43	4.29 ± 0.63	*t*(198) = 1.49	0.139
	Female	157	4.13 ± 0.62		
Age Group	21–25 years	51	4.09 ± 0.57	*F*(3, 196) = 2.96	0.034 *
	26–30 years	50	4.12 ± 0.66		
	31–40 years	58	4.36 ± 0.48		
	>40 years	41	4.04 ± 0.75		
Years of Experience	1–3 years	69	4.06 ± 0.71	*F*(3, 196) = 1.63	0.183
	3–6 years	46	4.22 ± 0.62		
	6–10 years	36	4.33 ± 0.47		
	>10 years	49	4.15 ± 0.58		
Educational Qualification	Bachelor’s Degree	117	4.17 ± 0.68	*F*(3, 196) = 1.24	0.295
	Pharm. D.	20	4.38 ± 0.52		
	Master’s Degree	35	4.13 ± 0.52		
	PhD	28	4.03 ± 0.51		
Years of Practice in Community Pharmacy	1–3 years	73	4.12 ± 0.67	*F*(3, 196) = 2.30	.078
	3–6 years	51	4.36 ± 0.59		
	6–10 years	26	4.13 ± 0.65		
	>10 years	50	4.06 ± 0.53		

*Note.* Independent-samples *t*-tests and one-way tests. * p = < *0.05*.

**Table 9:** Adjusted Between-Group Comparisons of Post-Intervention Outcomes and Pre–Post Changes (*n* = 200).

Analysis	Outcome	Intervention Adjusted Mean (95% CI)	Control Adjusted Mean (95% CI)	HC3-Adjusted Difference (95% CI)	Robust *t* (df)	*p*	Partial η^2^
Baseline-adjusted post-test comparison	Total knowledge ^1^	14.57 (14.12, 15.01)	7.84 (7.35, 8.33)	6.73 (6.13, 7.33)	22.22 (192)	<0.001 *	0.720
	Attitude ^2^	4.09 (3.92, 4.26)	3.51 (3.37, 3.65)	0.579 (0.393, 0.765)	6.14 (192)	<0.001 *	0.164
Adjusted post-test comparison	Satisfaction ^3^	4.35 (4.22, 4.47)	4.04 (3.90, 4.19)	0.304 (0.122, 0.487)	3.29 (194)	0.001 *	0.053
Adjusted pre–post change comparison	Total knowledge ^4^	7.52 (7.05, 8.00)	0.49 (−0.05, 1.03)	7.04 (6.37, 7.71)	20.77 (194)	<0.001 *	0.690
	Attitude ^4^	0.161 (0.049, 0.273)	0.070 (−0.056, 0.196)	0.091 (−0.061, 0.242)	1.18 (194)	0.238	0.007

*Note*. HC3 heteroskedasticity-consistent standard errors, confidence intervals, and *p* values are reported for the adjusted between-group differences. ^1^ Post-intervention total knowledge was adjusted for baseline knowledge, gender, and age group. Because the group × baseline-knowledge interaction was significant, *F*(1, 192) = 32.62, *p* < 0.001, partial η^2^ = 0.145, we estimated the comparison conditionally at the pooled baseline-knowledge mean of 7.405. ^2^ Post-intervention attitude was adjusted for baseline attitude, gender, and age group. Because the group × baseline-attitude interaction was significant, *F*(1, 192) = 8.08, *p* = 0.005, partial η^2^ = 0.040, the comparison was estimated conditionally at the pooled baseline attitude mean of 3.5598. ^3^ Satisfaction was measured only after the intervention and was therefore adjusted for gender and age group without a baseline satisfaction covariate. ^4^ Change scores were calculated as post-test minus pre-test scores and adjusted for gender and age group. Positive values indicate improvement. * p ≤ 0.05.

## Data Availability

The data presented in this study are available on request from the corresponding author. The data are not publicly available due to privacy and ethical restrictions.

## Supplementary Materials

The following supporting information can be downloaded at https://www.mdpi.com/article/10.3390/healthcare14193260/s1, Table S1: Educational intervention curriculum. Table S2: Intervention questionnaire. Table S3: Satisfaction questionnaire.

## Abbreviations

The following abbreviations are used in this manuscript:

DKT	Diabetes knowledge test to measure knowledge and a modified Diabetes Attitude Scale
DAS-3	Diabetes Attitude Scale, version 3
KA	Knowledge and attitude
MDRTC	Michigan Diabetes Research and Training Center
SPSS	Statistical Package for the Social Sciences
T2DM	Type 2 Diabetes Mellitus
