# Supplementary material for: Evaluation of a Structured Educational Program for Community Pharmacists’ Knowledge, Attitudes, and Satisfaction Toward Diabetes Patient-Centered Care: A Non-Randomized Quasi-Experimental Study
Source: Healthcare (Basel). 2026 Oct 1;14(19):3260. doi: 10.3390/healthcare14193260 (PMC13665090; doi:10.3390/healthcare14193260)
Supplement: Supplementary file 1 [file healthcare-14-03260-s001.zip › healthcare-4515069-supplementary.pdf]

**Table S1. Educational intervention curriculum.**

| Lesson Title | Contents | Time Allocation |
|---|---|---|
| **1. Introduction to Diabetes & Role of Pharmacists** | - Overview of diabetes types (Type 1, Type 2, gestational)<br>- Risk factors, diagnostic criteria, and normal lab values<br>- Role of community pharmacists in early detection and referral | 10 minutes |
| **2. Patient-Centered Care in Diabetes** | - Principles of patient-centered care<br>- Effective communication and active listening<br>- Motivational interviewing<br>- Cultural and contextual considerations in Palestine | 15 minutes |
| **3. Lifestyle and Non-Pharmacological Management** | - Lifestyle interventions: physical activity, weight management<br>- Nutrition basics for diabetic patients<br>- Smoking cessation and behavior change support | 10 minutes |
| **4. Oral Anti-Diabetic Medications** | - Mechanisms, indications, and counseling for:<br>• Metformin, Sulfonylureas, DPP-4 inhibitors, SGLT2 inhibitors, TZDs<br>- Adherence strategies and side effect management | 10 minutes |
| **5. Insulin and Injectable Therapies** | - Types of insulin and GLP-1 agonists<br>- Insulin initiation and titration<br>- Hypoglycemia prevention and management<br>- Injection technique and patient education | 10 minutes |
| **6. Managing Complications and Special Populations** | - Chronic complications: neuropathy, nephropathy, retinopathy, diabetic foot care<br>- Special populations: elderly, pregnant women, adolescents<br>- Referral and follow-up | 10 minutes |
| **7. Case-Based Discussion and Interactive Q&A** | - Case scenarios for counseling, medication selection, and lifestyle advice<br>- Group discussion and real-life problem-solving<br>- Open Q&A session | 10 minutes |

**Table S2. Intervention questionnaire**

## Diabetes Education and Awareness Session

*Demographic Data*

| Question | Options |
|---|---|
| Gender | [ ] Male<br>[ ] Female |
| Age Group | [ ] 21-25<br>[ ] 26-30<br>[ ] 31-40 |

| | |
|---|---|
| | [ ] 41-50<br>[ ] More than 50 |
| Years of Experience | [ ] 1-3 years<br>[ ] 3-6 years<br>[ ] 6-10 years<br>[ ] More than 10 years |
| Educational Qualification | [ ] Bachelors in Pharmacy (B.Pharm)<br>[ ] Doctor of Pharmacy (Pharm.D.)<br>[ ] Master's degree in Pharmacy<br>[ ] PhD<br>[ ] Others (specify): |
| Years of Practice in Community Pharmacy in Palestine | [ ] 1-3 years<br>[ ] 3-6 years<br>[ ] 6-10 years<br>[ ] More than 10 years |

**Diabetes Knowledge Test**

**Instruction: Please circle the correct answer.**

a. Nutrition and Monitoring

| Question | Options |
|---|---|
| The diabetic diet is | [ ] The way most Palestinian people eat<br>[ ] A healthy diet for most people<br>[ ] Too high in protein for most people |
| Which of the following is highest in carbohydrates? | [ ] Bar B Q Chicken<br>[ ] Yoghurt<br>[ ] Rice<br>[ ] Oranges |
| HbA1c measures your average blood glucose level for the past | [ ] Day<br>[ ] Week<br>[ ] 6-12 weeks<br>[ ] 6 months |
| For a diabetic patient in good control, what effect does exercise have on blood glucose? | [ ] Lowers the blood glucose<br>[ ] Increases the blood glucose<br>[ ] Has no effect |
| Which problem is usually not associated with diabetes? | [ ] Kidney disease<br>[ ] Eye diseases<br>[ ] Nerve disease<br>[ ] Lung diseases |
| What effect will an infection most likely have on blood glucose? | [ ] Lowers the blood glucose<br>[ ] Increases the blood glucose<br>[ ] Has no effect |
| Which should not be used to treat low glucose levels? | [ ] Increases the blood glucose<br>[ ] Has no effect<br>[ ] Lowers the blood glucose (Incorrect) |

b. Oral Pharmacotherapy

| Question | Options |
|---|---|
| Vitamin B12 deficiency is associated with which diabetic medicine? | [ ] Metformin<br>[ ] Glipizide<br>[ ] Linagliptin |

| | |
|---|---|
| Which diabetic medicine does not cause weight gain? | [ ] Glimepiride<br>[ ] Glipizide<br>[ ] Liraglutide |
| The preferred treatment for type 2 diabetes during pregnancy is | [ ] Insulin<br>[ ] Glipizide<br>[ ] Canagliflozin<br>[ ] Liraglutide |

c. Knowledge about Insulins

| Question | Options |
|---|---|
| Sign of diabetic ketoacidosis is | [ ] Shaking of body<br>[ ] Sweating<br>[ ] Vomiting<br>[ ] Low blood glucose |
| If you are sick with the flu, you should | [ ] Take less insulin<br>[ ] Drink fewer fluids<br>[ ] Eat more proteins<br>[ ] Test blood glucose more often |
| If you have rapid-acting insulin, you are most likely to have a low blood glucose reaction in | [ ] Less than 2 hours<br>[ ] 3-5 hours<br>[ ] 6-12 hours<br>[ ] More than 13 hours |
| Which insulin is considered basal insulin? | [ ] Insulin Aspart<br>[ ] Insulin glargine<br>[ ] Insulin lispro<br>[ ] Regular insulin |

d. Complications of Diabetes

| Question | Options |
|---|---|
| The best way to take care of diabetes feet is to | [ ] Look at the feet and wash them every day<br>[ ] Massage them with alcohol every day<br>[ ] Soak them for one hour each day<br>[ ] Wear a large size shoe |
| Numbness and tingling may be symptoms of: | [ ] Kidney disease<br>[ ] Nerve disease<br>[ ] Eye disease<br>[ ] Liver disease |
| High blood glucose may be caused by: | [ ] Not enough insulin<br>[ ] Skipping meals<br>[ ] Delaying your snacks<br>[ ] Skipping your exercise |

*Diabetes Attitude Scale*

| Statement | Strongly Agree | Agree | Neutral | Disagree | Strongly Disagree |
|---|---|---|---|---|---|
| Pharmacists should be trained to communicate well with diabetic patients | | | | | |
| Daily diabetes care impacts patients' lives significantly | | | | | |
| Pharmacists should set treatment goals with patients | | | | | |
| Counseling about side effects is crucial | | | | | |

| | |
|---|---|
| Type 2 Diabetes does not require insulin | |
| Blood sugar testing is unnecessary for Type 2 Diabetes patients | |
| Older patients with diabetes usually do not get complications | |
| Keeping blood sugar close to normal prevents complications | |
| Type 2 diabetes patients benefit from controlling their blood sugars | |
| Diabetes care decisions should be made by the person with diabetes | |
| The person with diabetes is the most important team member | |
| Pharmacists should assist patients in making informed care decisions | |

**Table S3. Satisfaction questionnaire**

**Instructions:** Please answer the following questions based on your recent participation in our diabetes management training program. Your feedback is invaluable and will help improve future training sessions.

**Demographic Information:**

- **Age:** ________
- **Years of Experience in Community Pharmacy:** ________
- **Gender:** ________
- **Location of Practice:** ________

| **Section 1: General Satisfaction** | **Definitely Yes** | **Yes** | **Not Sure** | **Not Quit** | **Definitely No** |
|---|---|---|---|---|---|
| The objectives of the diabetes management training program were clearly defined and met. | | | | | |
| The content of the training was relevant to my practice. | | | | | |
| The training content was organized and easy to follow. | | | | | |
| The time allocated for the training program was realistic and sufficient. | | | | | |
| **Section 2: Impact on Practice** | **Definitely Yes** | **Yes** | **Not sure** | **Not Quit** | **Definitely No** |
| The training improved my knowledge about diabetes prevention and management. | | | | | |
| Hands-on training improved my skills in patient counseling for diabetes. | | | | | |
| Lecturers communicated information clearly and effectively. | | | | | |

| | |
|---|---|
| The educational program has motivated me to participate in further diabetes-related education | |
